# Protein tandem repeats that produce frameshifts can generate new structural states and functions

**DOI:** 10.1111/febs.70273

**Published:** 2025-09-24

**Authors:** Zarifa Osmanli, Gudrun Aldrian, Jeremy Leclercq, Theo Falgarone, Santiago M. Gómez Bergna, Denis N. Prada Gori, Andrew V. Oleinikov, Ilham Shahmuradov, Andrey V. Kajava

**Affiliations:** ^1^ CRBM Université de Montpellier, CNRS France; ^2^ Institute of Biophysics Ministry of Science and Education of Azerbaijan Republic Baku Azerbaijan; ^3^ Institute of Biotechnology and Molecular Biology (IBBM, UNLP‐CONICET), Faculty of Exact Sciences National University of La Plata Buenos Aires Argentina; ^4^ Laboratory of Bioactive Compound Research and Development (LIDeB), Faculty of Exact Sciences National University of La Plata Buenos Aires Argentina; ^5^ Department of Biomedical Science, Charles E. Schmidt College of Medicine Florida Atlantic University Boca Raton FL USA; ^6^ Genetic Resources Institute Ministry of Science and Education of the Republic of Azerbaijan Baku Azerbaijan

**Keywords:** alphafold, codon usage, frameshifting, large‐scale analysis, tandem repeat proteins

## Abstract

The genetic code uses three‐nucleotide units to encode each amino acid in proteins. Insertions or deletions of nucleotides not divisible by three shift the reading frames, resulting in significantly different protein sequences. These events are disruptive but can also create variability important for evolution. Previous studies suggested that the genetic code and gene sequences evolve to minimize frameshift effects, maintaining similar physicochemical properties to their reference proteins. Here, we focused on tandem repeat sequences, known as frameshift hotspots. Using cutting‐edge bioinformatics tools, we compared reference and frameshifted protein sequences within tandem repeats across 50 prokaryotic and eukaryotic proteomes. We showed that, in contrast to the general tendency, frameshifts within these regions, especially with short repeats, lead to a significant increase in hydrophobicity and arginine content. Additionally, the frameshifts, particularly in short tandem repeats, rearrange transmembrane regions, potentially converting soluble proteins into membrane proteins and vice versa. Given their occurrence in rapidly evolving, essential proteins, such changes may promote rapid adaptability. Our large‐scale alphafold modeling suggested that frameshift events can generate novel structures and functions, enabling the synthesis of multiple protein variants within the same coding region. Overall, frameshifts cause more drastic changes in tandem repeat sequences compared to non‐repetitive sequences and therefore can be a primary cause of altered functions, cellular localization, and the development of various pathologies.

## Graphical abstract

We explored an alternative protein structure landscape by analyzing amino acid sequences from frameshifted tandem repeats—regions prone to frameshifts. These frameshifts, especially in short repeats, lead to more drastic changes than in non‐repetitive regions, often altering structure, function, localization, and potentially contributing to disease. Such frameshifts can also create novel protein variants from the same coding region.

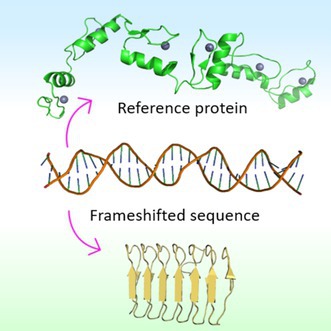

**Abbreviations**
3D: three‐dimensional
AA: amino acid
AR: aggregation‐prone region
CDS: coding sequence
EAR: exposed aggregation‐prone regions
IDR: intrinsically disordered region
SLiM: short linear motif
TM: transmembrane
TR: tandem repeat

## Introduction

Genetic information flows from DNA through messenger RNA into protein [1]. The genetic code, which translates information from the ‘language’ of nucleic acids to that of proteins, follows a triplet nature, where three nucleotides encode a single amino acid. Due to the triplet nature of the genetic code, an insertion or deletion of a number of nucleotides that is not divisible by three can shift the reading frame, resulting in significantly different protein sequences. Besides the frameshifts induced by the indels, a shifting process can also occur on ribosomes when they translate the same mRNA into different polypeptide chains [2, 3, 4, 5]. This Programmed Ribosomal Frameshifting serves to increase the protein‐coding capacity of genomes [6, 7] and to downregulate protein translation in all kingdoms of life [8, 9]. Splicing of exons in different frames also enriches the diversity of functional genes in eukaryotic organisms [10].

The frameshift event, in which a minor nucleotide‐level alteration can result in profound protein‐level changes, appears to be exceptionally efficient for the generation of the variability required for the process of evolutionary selection. The frameshift mutations are mostly known for their disruptive nature [11]. They typically result in significantly modified protein sequences, often with premature stop codons that, in turn, lead to nonfunctional or potentially harmful products [12]. Frameshifts are frequently associated with various health disorder variants, including cancer [13]. Thus, experimental studies increasingly show that frameshifted proteins can be expressed, often via alternative promoters and other gene expression components. However, these approaches do not capture the full spectrum of possibilities, as many mechanisms enabling the expression of proteins in alternative reading frames remain unknown and a much larger portion of DNA sequences exists within an evolutionary pool yet to be explored.

To address this, large‐scale bioinformatics approaches, enabled by high‐quality genome data and advances in proteomics, offer a broader, system‐level view. These methods move beyond isolated cases, revealing hidden protein‐coding potential that may, under the right conditions, become biologically functional. Several such studies suggested that the genetic code may be optimized during evolution to minimize the effects of errors introduced by frameshifting [14, 15, 16]. Furthermore, it has been demonstrated that several characteristics of protein sequences, including their hydrophobicity profiles and intrinsic disorder profiles, maintain similarity in the corresponding frameshifted sequences. This result suggests that frameshifting could serve as an evolutionary mechanism for generating new proteins with significantly distinct sequences while maintaining similar physicochemical properties to their parent proteins [17].

In this work, we focused on a comparative proteome‐wide analysis of reference proteins and their frameshifted sequences containing tandem repeats (TRs). We studied five groups of TRs, classifying them according to the length of their repetitive units, in agreement with an established structural classification [18]. There are several compelling reasons to gain a more profound understanding of the structural and functional implications of frameshifts that can occur within TRs. First, proteins containing TRs are widespread in genomes, appearing in nearly one‐third of human proteins and in as many as half of the proteins from *Dictyostelium discoideum* or *Plasmodium falciparum* [19, 20]. Second, the frequency of frameshift events has been shown to increase in TRs having short repetitive units of 1 to 3 residues [21, 22]. As concerns longer repeats, they originate from tandem duplication [23]. The duplication, coupled with frameshift mutations, is proposed to serve as a common mechanism for generating functional innovations in proteins [24]. Third, TRs, particularly those with short repeats, often exhibit a pronounced bias in amino acid compositions, resulting in a heightened potential for molecular interactions due to the elevated local concentration of specific physicochemical properties such as hydrophobicity, charge, or flexibility [25]. The occurrence of frameshift mutations within these TRs has the potential to entirely alter their sequences, resulting in markedly different structural and functional characteristics, with the amplitude of these changes being notably higher than in aperiodic protein sequences. Recognizing the significance of this phenomenon and the absence of systematic studies, our comprehensive proteome‐wide analysis aims to investigate the potential changes in protein sequence, structure, and function resulting from frameshifts within TRs.

**Fig. 1:**
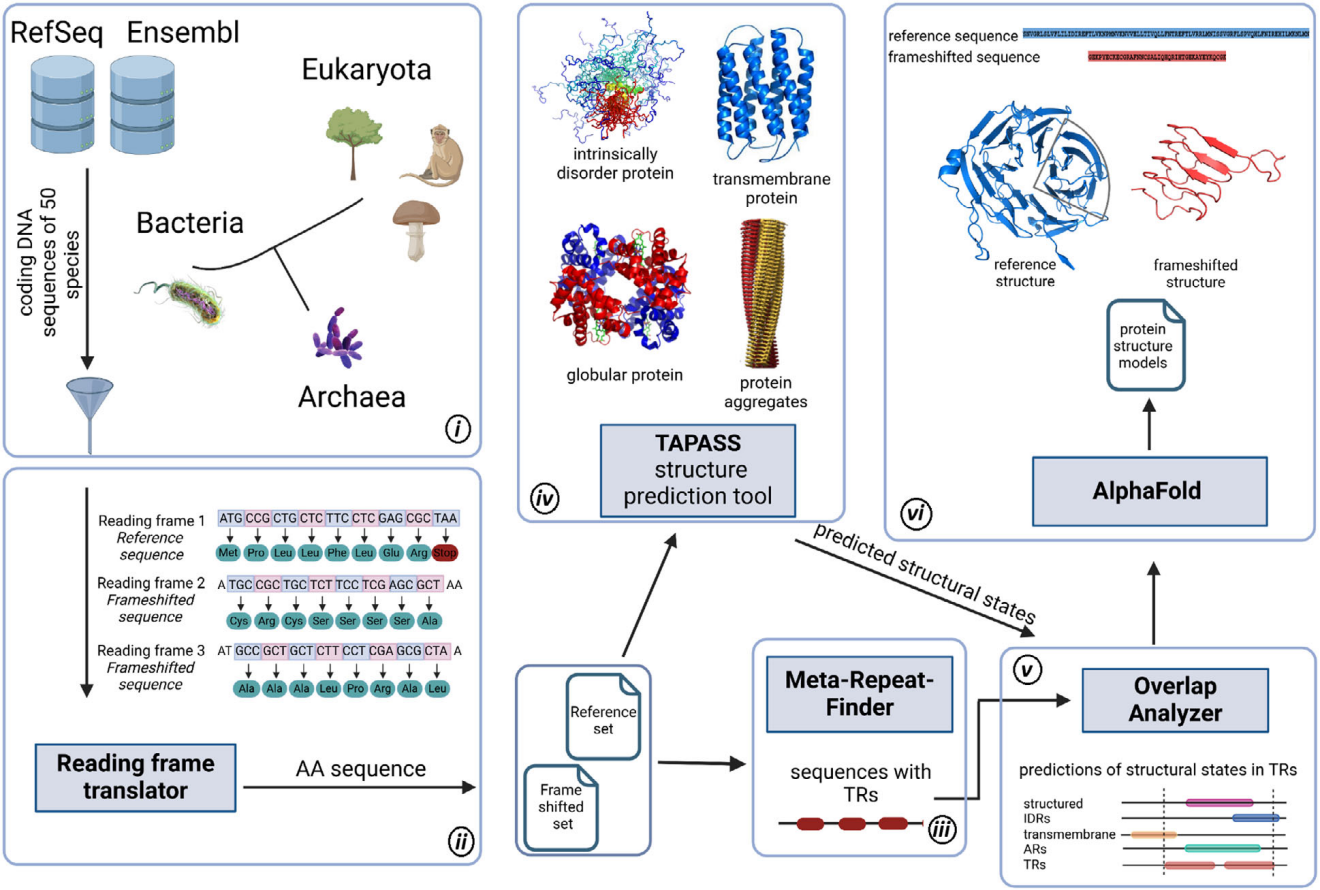
Flowchart of the main steps in tandem repeat (TR) analysis: (i) Selection of well‐annotated prokaryotic and eukaryotic genomes; (ii) Generation of +1/−1 frameshifted amino acid sequences (frame 2/frame 3 in the figure) using the ‘Reading Frame Translator’ script; (iii) Detection of TRs in reference and frameshifted sequences with Meta‐Repeat‐Finder; (iv) Structural annotation of amino acid sequences using tapass [26] and other tools (amino acid composition, repeat motifs, conformational order/disorder, aggregation, membrane association); (v) Mapping annotations to TR regions by ‘Overlap Analyzer’ script; (vi) 3D structure prediction of TRs with alphafold [27].

Our workflow included (a) large‐scale detection of TRs in both reference and frameshifted sequences from well‐studied prokaryotic and eukaryotic organisms; (b) classification of TRs based on repeat length, given its known influence on structural properties; (c) comparative analysis of the structural characteristics of TRs from reference and frameshifted sequences, including amino acid composition, dominant repeat motifs, propensity to form structured domains or remain intrinsically disordered, aggregation potential, and likelihood of membrane association. Additionally, the high accuracy of alphafold allowed us to predict three‐dimensional (3D) structures for proteins containing frameshifted TRs (Fig. 1 and Fig. S1). This analysis enabled us to assess how frameshifting alters TR structural and functional properties and to explore potential evolutionary constraints on both reference and frameshifted sequences.

## Results

### Distribution of TRs in reference and frameshifted sequences

**Fig. 2:**
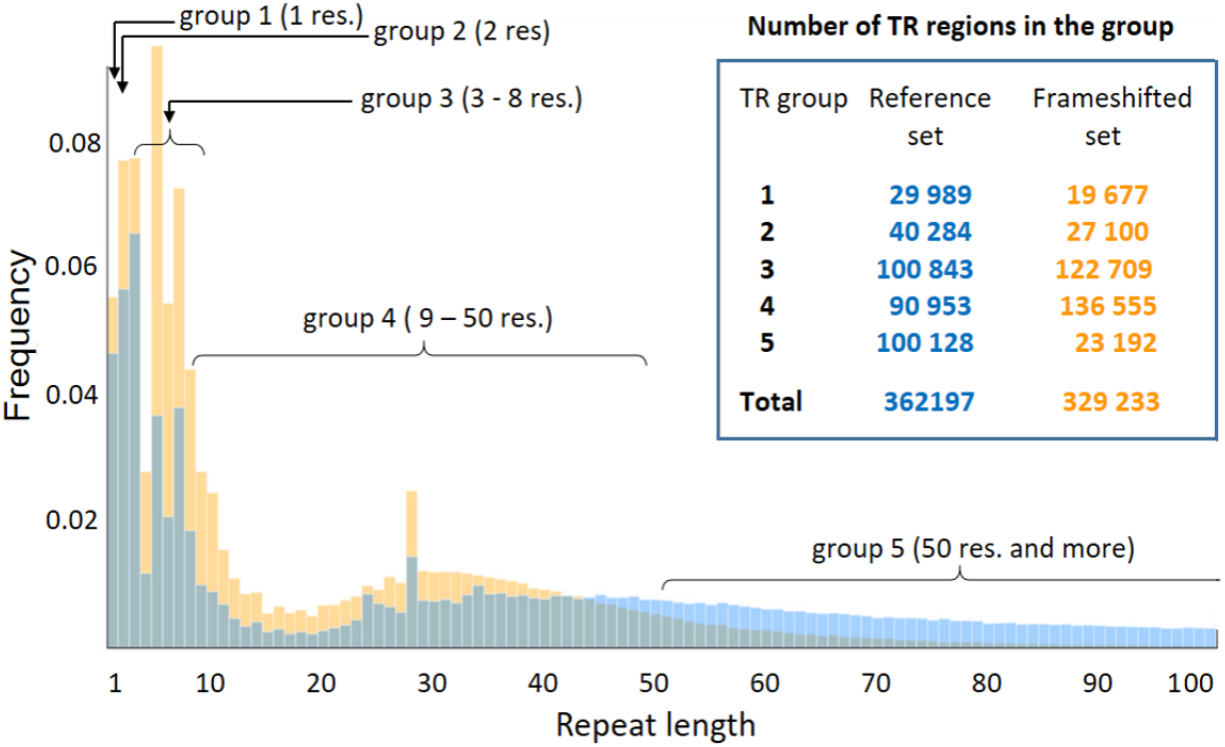
The frequency of tandem repeats (TRs) depends on their repeat length. The frequencies are computed by dividing the number of TRs with a specific repeat length by the total number of TRs within either the reference (blue) or frameshifted (orange) sets. The histograms depict the frequencies of TRs up to the repeat length of 100 amino acid residues. TRs are subdivided into five groups based on the length of their repeats, and the inset on the right side of the histogram contains a table showing the number of TRs in each group. In the figure, ‘res’, stands for amino acid residues.

Since amino acid periodicity is encoded at the DNA level, TRs in reference proteins often persist in frameshifted sequences. For example, in *Sclerotinia sclerotiorum*, 33.5% of TR‐containing proteins also show TRs in alternative frames. Frameshifted AA sequences are usually shorter than the reference proteins due to frequent stop codons. Therefore, certain TR regions disappear in the frameshifted set. We found that 16.60% of reference sequences and 9.45% of sequences with −1 and +1 frame shifts contain TRs. In terms of TR regions, the reference and frameshifted sets contain 362 197 and 329 233 regions, respectively (Fig. 2). Both sets show similar TR frequency patterns, with short repeats being the most prevalent (Fig. 2). Similarly to the reference set, frameshifted TRs are enriched in the 5‐ and 7‐residue repeats. We also observed a peak at 28‐residue repeat in both sets, which in the reference set corresponds to the Zn‐finger repeats. However, long repeats (> 50 residues) are rare in frameshifted sequences, likely due to their shorter overall length. Frameshifted TR‐containing sequences average 130 AA in length with 19‐residue repeats, compared to 807 AA and 60‐residue repeats in the reference set.

### Subdivision of the analyzed TRs into groups based on the repeat length

TRs exhibit distinct structural and functional properties depending on their repeat length [18]. Additionally, the likelihood of TRs adopting disordered conformations increases as repeat length decreases [20, 28]. Thus, for our analysis, it was instrumental to subdivide TRs into several groups depending on repeat length. While these groups are similar to the Classes in the structural classification [18], they feature non‐overlapping repeat length ranges—a distinction that facilitates the current analysis. Group 1 includes homorepeats with single‐residue units, and Group 2 comprises two‐residue repeats; both fall under Class I of the structural classification. Group 3 includes repeats of 3 to 8 residues, corresponding mainly to Class II and partially to Class III. Group 4 consists of repeats ranging from 9 to 50 residues, aligning primarily with Class III and to a lesser extent with Classes IV and V. Group 5 includes repeats longer than 50 residues, corresponding mostly to Class V and partly to Class IV. Figure 2 displays the counts of TRs in each group.

### AA composition of TR regions in the reference and frameshifted sequences

**Fig. 3:**
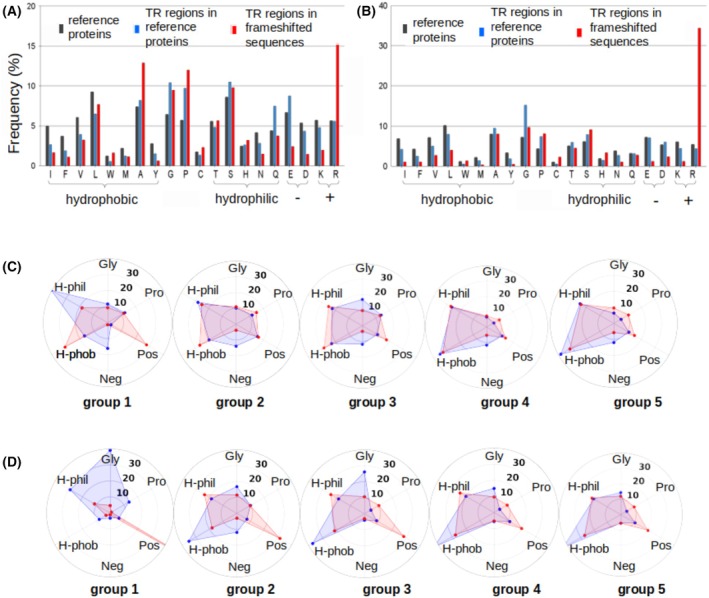
Histograms of amino acid (AA) composition of the reference and frameshifted sets of tandem repeats. (A) Eukaryotes. (B) Prokaryotes. AA frequencies (%) in all analyzed proteins (black bars), in tandem repeat regions of proteins (blue bars), and in frameshifted sequences of these tandem repeat regions (red bars). AAs are denoted by a one‐letter code. Hydrophobic residues are Ile, Phe, Val, Leu, Trp, Met, Ala, Tyr; hydrophilic residues are Ser, Thr, His, Asn, Gln; negatively charged are Glu, Asp; positively charged are Lys, Arg. Hereinafter, AA frequency of a specific AA = Total number of a specific AA in the set/Total number of all AAs in the set. Polygon graphs of AA composition (%) of the five tandem repeat groups in eukaryotes (C) and prokaryotes (D). Gly, glycine; H‐Phil, hydrophilic; H‐Phob, hydrophobic; Neg, negatively charged; Pos, positively charged residues; Pro, proline. Polygons of reference proteins and frameshifted sequences are in blue and red, respectively. For clarity of the graphs, the cysteine residue is not shown because it is rare in both sets (reference and frameshift).

We compared the amino acid composition of TRs in reference and frameshifted sequences, analyzing eukaryotic and prokaryotic proteins separately (Fig. 3A,C, and Tables S1 and S2). In eukaryotes, frameshifted sequences show a marked increase in positively charged residues, particularly arginine (Arg), and a decrease in negatively charged ones (Fig. 3A). Hydrophobicity also rises due to more apolar residues, especially alanine (Ala), and fewer polar ones, notably glutamine (Gln). In prokaryotes, frameshifted TR regions also show elevated Arg levels (Fig. 3B), but unlike in eukaryotes, overall hydrophobicity decreases. Overall, a consistent trend in both domains is a notable increase in positive charge in frameshifted TRs compared to reference sequences.

We compared the amino acid (AA) composition of TR regions across five groups, noting the following key differences between reference and frameshifted sequences (Fig. 3C,D, Tables S1 and S2).

#### Group 1

In the reference proteins of eukaryotes, the homorepeats are typically hydrophilic, with a higher prevalence of negative charges compared to positive ones (Fig. 3C). They are enriched (in order of increasing occurrence) in Gln, Ser, Glu, Pro, Ala, and Gly (Table S1 and Fig. S2). In contrast, frameshifted sequences are more hydrophobic, mostly due to increased Ala and Leu, with a sharp decline in Gln and negatively charged residues, and a marked rise in positively charged residues, namely Arg (Fig. 3A,C). In prokaryotes, this increase in Arg was observed to an even greater extent (Fig. 3D). Unlike eukaryotes, prokaryotic frameshifted homorepeats, similarly to the reference ones, have a very low percentage of hydrophobic residues.

The surge in poly‐Arg is likely due to Arg being encoded by six different codons, compared to most amino acids, which are encoded by fewer codons. This increases the likelihood of Arg appearing in frameshifted sequences. However, codon count alone does not fully explain the increase, as the other amino acids with six codons, such as Ser and Leu, do not show a similar rise. A key factor appears to be that three Arg codons in alternative reading frames correspond to Glu, Ser, and Ala, amino acids frequently found in reference homorepeats (Figs S3–S5).

#### Group 2

In eukaryotes, two‐residue repeats in both reference and frameshifted sets are enriched in Ser, Pro, Arg, and Gly (Table S1). While reference TRs show balanced positive and negative charges, frameshifting causes a drastic increase in positive charge due to a reduction in negatively charged residues (Fig. 3C). A moderate increase in hydrophobicity is also observed in frameshifted TRs. In prokaryotes, the charged residue pattern follows the same trend, but the hydrophobicity trend is reversed (Fig. 3D).

#### Group 3

In eukaryotes, the most frequent residue in the reference set is Gly, followed by Pro (Fig. 3C,D). This can be explained by the presence of collagen sequences in this group. Gly is also the most frequent in prokaryotes. The frameshifted sequences of both prokaryotes and eukaryotes are enriched in Arg. The hydrophobicity of TRs in eukaryotic sequences is similar to that of their frameshifted sequences and even higher in the reference proteins of prokaryotes.

#### Groups 4 and 5

**Fig. 4:**
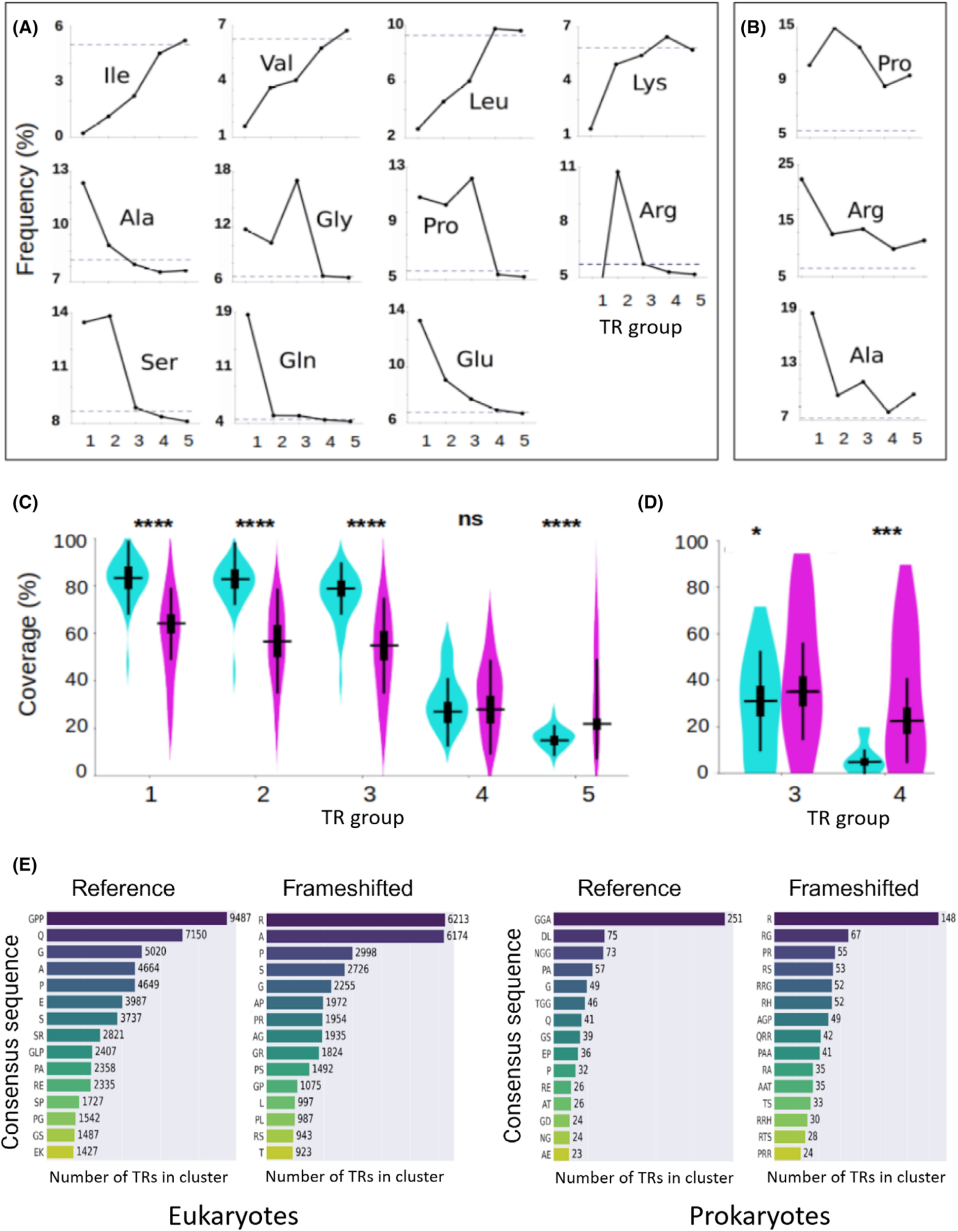
Line graphs of the most prominent changes in amino acid (AA) frequencies depending on the tandem repeat (TR) groups in eukaryotic proteins. (A) Reference sequences. (B) Frameshifted sequences. Broken lines denote the average percentage of a given AA in reference proteomes. (C) Coverage of Intrinsically Disordered Regions (IDRs) within TR regions (percentage of AAs in TRs, which have unstructured conformation) of proteins in eukaryotic organisms and (D) in prokaryotes. The results for reference proteins and frameshifted sequences are shown in blue and magenta, correspondingly, and for each TR group separately. The *x*‐axis labels (1–5) indicate the TR group numbers. In the violin plots, the horizontal line marks the median, and the thick vertical bar denotes the interquartile range (25th–75th percentiles). The observed variance is statistically significant between reference and frameshifted sets performed by the two‐sided *T*‐test. ****, *** and * mean *P*‐value < 0.0001, *P*‐value < 0.001 and *P*‐value < 0.05, respectively. ns means non‐significant. (E) Top clusters of TRs in reference and frameshifted sequences of eukaryotic and prokaryotic organisms. AAs are denoted by a one‐letter code.

TRs have similar amino acid compositions in both eukaryotes and prokaryotes, so the following conclusions apply to both types of organisms. Unlike short repeats, those longer repeats exhibit increased hydrophobicity in reference proteins compared to the frameshifted sequences (Fig. 3C,D). At the same time, the reference TRs continue to be predominantly composed of negatively charged residues, whereas the frameshifted TRs primarily contain positively charged ones. Overall, the amino acid composition of reference TRs aligns more closely with the average composition of the analyzed proteomes (Fig. 4A). In frameshifted TRs, Arg remains the most frequent residue (Tables S1 and S2).

Thus, generally, frameshifted TRs across all groups contain more positively and fewer negatively charged residues than reference TRs (Fig. 3C,D). They have a drastic drop in the frequency of polar Gln, negatively charged residues, and a strong prevalence of the positively charged Arg (Fig. 4A,B, Tables S1 and S2). Frameshifted TRs show higher hydrophobicity in short repeats but lower hydrophobicity in longer ones.

### Typical repeats detected by clustering

We clustered the repeat motifs (see Materials and methods). Clusters that comprise the greatest number of TRs sharing identical consensus sequences primarily consist of homorepeats (Fig. 4E). In eukaryotes, as expected, the top‐ranked repeat motifs of the reference proteins obtained by the clustering correspond to the same most frequent AAs of the homorepeats revealed by AA composition analysis (Gln (7150 times), Gly (5020), Ala (4664), Pro (4649), Glu (3987), and Ser (3737)). The clustering of two‐residue repeats from group 2 showed that the most frequent pairs are Ser‐Arg (2821), Pro‐Ala (2358), Arg‐Glu (2335), Ser‐Pro (1727), Pro‐Gly (1542), Gly‐Ser (1487), and Glu‐Lys (1427). This is generally in agreement with the AA composition of the two‐residue repeats, which are enriched in Ser, Pro, Arg, Gly, Glu, and Ala (Table S1). At the same time, not all combinations of the most frequent AAs are present among the top‐ranked repeats from group 2. For example, the negatively charged Glu‐Ser, Glu‐Gly, Glu‐Ala, and Glu‐Pro are not at the top of the list, in contrast to the positively charged TR formed by Ser‐Arg and Arg‐Gly, with Ser‐Arg being the most frequent motif in group 2. This suggests that Arg‐containing two‐residue repeats play an important functional role in the reference proteins. Group 3 exhibits an abundance of Gly and Arg, with the Gly‐Pro‐Pro motif representing the largest cluster (9487) not only within this group but also across all the groups (Fig. 4E). This can be explained by the widespread occurrence of the collagen sequences in the reference proteins of eukaryotes. The clusters of TRs from group 4 and group 5 have significantly fewer members, and as a result, even the top‐ranked TR sequences cannot be regarded as typical TR motifs.

For frameshifted eukaryotic sequences, the top‐ranked repeat motifs of group 1 are homorepeats of Arg (6213), Ala (6174), Pro (2998), Ser (2726), and Gly (2255) (Fig. 4E). The most significant distinction from the reference proteins is the emergence of positively charged Arg, coupled with the absence of negatively charged Glu and polar Gln. TRs from group 2 follow this trend, having apolar TRs with small side chains (Ala‐Pro and Ala‐Gly) and positively charged ones (Arg‐Pro and Arg‐Gly) as the most typical motifs. The TRs from group 3 are not among the 15 top‐ranked motifs (Fig. 4E). In comparison with the reference set, these TRs lack collagen‐like motifs but primarily consist of positively charged residues and small‐sized residues.

In prokaryotes, TR sets are significantly smaller. Within the reference TRs, homorepeats from group 1 are less common, with only three of them (Gly, Gln, and Pro) appearing in the 15 top‐ranked motifs (Fig. 4E). In group 2, the most prevalent motifs consist of combinations of small‐sized residues along with negatively charged ones, such as Asp‐Leu, Pro‐Ala, Gly‐Ser, and Glu‐Pro. Surprisingly, one of the most frequent motifs belongs to group 3. These motifs (Gly‐Gly‐Ala, Gly‐Gly‐Asn, Gly‐Gly‐Thr) are commonly observed in bacterial cell surface proteins from the PE family [29]. The typical TR motifs of the frameshifted sequences significantly differ from the motifs of the reference proteins, with a pronounced prevalence of Arg homorepeats (Fig. 4E). Additionally, other frequently occurring motifs belong to group 2 and group 3 (such as Arg‐Gly, Arg‐Pro, Arg‐Ser, Arg‐Arg‐Gly, and Arg‐His), all of which are also notably enriched in Arg.

Thus, despite variation in TR motifs between prokaryotes and eukaryotes in the reference set, our clustering revealed in frameshifted sequences remarkable dominance of poly‐Arg, Pro‐Arg, and Gly‐Arg, regardless of the species, clearly indicating that the frameshifts favor the enrichment of positively charged Arg.

### Intrinsic disorder within TR regions

Intrinsically disordered regions (IDRs) are important attributes that enable the assessment of the structural states and functional roles of the analyzed proteins. We predicted IDRs by using IUPred [30] and observed that the coverage of IDRs in the reference TR regions from eukaryote and prokaryote sets is 57.20% and 21.24%, respectively. Notably, these percentages decrease to 47.26% in eukaryotes and increase to 31.86% in prokaryotes in the frameshifted sets. We observed that the shorter the repeats, the higher the disorder (Fig. 4C,D). For eukaryotes, the highest coverage of IDRs was observed in homorepeats from the reference set. The TRs from groups 2 and 3 (up to 8 residue‐long repeats) have a similarly high level of IDRs (Fig. 4C). The coverage of IDRs drops dramatically within TRs with repeats of more than 8 residues (groups 4 and 5). TRs of the frameshifted sequences from eukaryotes are less disordered than the reference ones in groups 1, 2, and 3. In groups 4 and 5, while the IDR coverage of the frameshifted TRs decreases in comparison with groups 1–3, it becomes slightly higher than that of the reference set.

In our prokaryotic set, the number of IDRs in the TR is insufficient to draw statistically valid conclusions for groups 1, 2, and 5. Therefore, Fig. 4D shows the results from groups 3 and 4 only, which had a sufficient number of IDRs to get statistically significant differences. In group 3, we noticed a slight increase in disorder within the frameshifted sets when compared to the reference ones, which stands in contrast to the trend observed in eukaryotes. The higher disorder in the frameshifted set than in the reference set is even more pronounced in group 4. A similar difference was observed in group 4 of eukaryotic species.

### In search of the 3D structures predicted by alphafold among frameshifted sequences

The majority of the frameshifted sequences are predicted to be intrinsically disordered [17]. In agreement with this result, we also predicted a large number of IDRs in the frameshifted TR sequences, particularly within groups 4 and 5 (Fig. 4C,D). Still, some of the sequences were predicted to be structured, prompting our further exploration to understand the atomic details of the formed 3D structures. For this purpose, we used the alphafold2 tool (see Materials and methods). In our previous analysis of protein isoforms, alphafold2 modeling suggested that a frameshift in one out of several exons of a gene frequently does not disrupt the overall protein structure [31]. Here, we analyzed the entire frameshifted TR regions in search of 3D structural models with high confidence scores. We selected a non‐redundant set of 14 031 TR‐containing frameshifted sequences, which were found at least two times across 29 eukaryotic species (see Materials and methods). Therefore, we called them ‘conserved’. Moreover, these sequences were predicted by tapass [26] to be structured. This set was used as input for our large‐scale alphafold2 modeling.

Among obtained AlphaFold2 structural models of frameshifted TR regions, only 475 had a high confidence score pLDDT ≥ 70. They were analyzed manually by pymol (http://www.pymol.org). A few of them, mostly Zn‐finger and ankyrin containing sequences, had a high confidence score of alphafold2 in the frameshifted sequences, while the corresponding reference sequences were predicted to be IDRs. This raises the question of whether the reference sequences containing ‘cryptic’ Zn‐fingers or ankyrin repeats are misannotated, and the frameshifted variants should, in fact, be included in the reference set instead. Our in‐depth analysis of such cases in the human proteome, aimed at verifying the existence of the reference proteins with cryptic Zn‐fingers or ankyrin repeats, revealed several instances where their presence was supported by mass spectrometry and/or RNA‐seq data (see Table S3). A previous mass‐spectral study also confirmed the expression of frameshifted proteins derived from reference Zn‐finger proteins [32]. At the same time, we observed that some reference proteins in public databases are annotated with a ‘predicted’ protein existence status, leaving open the possibility that some of these reference sequences may be misannotated.

blast analysis of these 475 frameshifted sequences against the SwissProt database (release 2025_01) identified 115 sequences with significant similarity (*E*‐value ≤ 10^−3^) to proteins from the same or different species. Some human frameshifted sequences showed high identity with different isoforms of the same protein, suggesting potential alternative frame usage in specific regions, such as exons or partial sequences. Among them are uncharacterized proteins that nonetheless share strong similarity with experimentally validated proteins from other species, suggesting potential functional relevance.

**Fig. 5:**
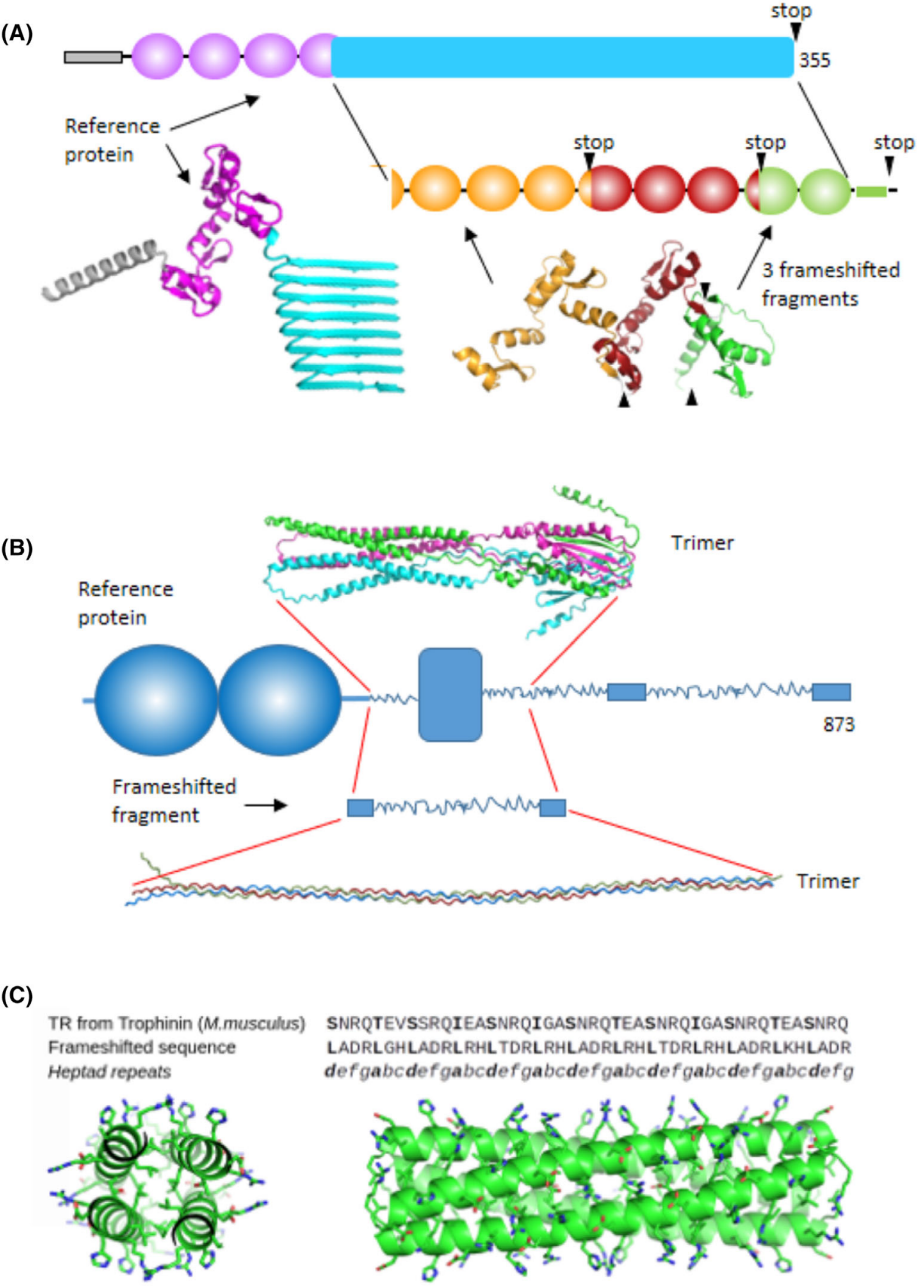
Comparative structural organization of proteins and their frameshifted variants. (A) Schematic representation of the structural arrangement in Zinc finger protein 781 (ZN781_HUMAN) and its frameshifted sequences. Black triangles denote the positions of stop codons. Oval shapes represent Zn‐finger domains, and a long blue rectangle indicates the location of the predicted solenoid structure. (B) Uncharacterized protein LOC563353 from *Danio rerio* (Q1LYI5_DANRE). In the reference protein, two N‐terminal domains have a well‐known structure, followed by a collagen‐like sequence and another domain with an unknown structure, and finally a long collagen‐like domain. The frameshifted sequence has a perfect collagen‐like pattern. (C) Trophinin (*Mus musculus*) E9Q160_MOUSE. alphafold2 prediction of the frameshifted sequence (pLDDT > 90). Axial and lateral projections of the tetrameric structure. Protein structure illustrations were generated using pymol (Schrödinger, LLC, New York, NY, USA).

Some of them represent interesting cases that deserve to be studied in depth. For example, Zinc finger protein 781 (ZN781_HUMAN) (Fig. 5A) has evidence of existence at the transcript level and has several orthologs in other species. Both the frameshifted sequence and the overlapping C‐terminal fragment of the reference protein are predicted to be structured. In the main frame, alphafold2 predicted solenoid structures, though with a low confidence score (pLDDT ~ 50) (Fig. 5A). At the same time, the frameshifted sequences have well‐recognized tandems of Zn‐finger domains (pLDDT ≥ 70) interrupted by three stop codons.

In another example, an uncharacterized protein LOC563353 from *Danio rerio* (Q1LYI5_DANRE) has several collagen‐like regions, suggesting that the overall 3D structure is trimeric (Fig. 5B). The protein has a long frameshifted sequence with a perfect collagen‐like pattern, which is highly likely to form a collagen triple helix. This frameshifted fragment overlaps with the region of the reference protein that is predicted to be structured. In this region of the main frame, AF3 predicted an α‐helical structure (pLDDT ~50) (Fig. 5B). The protein of *Danio rerio* also has several orthologs in other species.

Several frameshifted high‐quality annotated sequences have regions with well‐distinguished α‐helical coiled‐coil TRs. The corresponding reference sequences of most of them are predicted to have IDRs. However, in certain cases, such as in Trophinin (*Mus musculus*) E9Q160_MOUSE (Fig. 5C), alphafold2 models represent α‐helical coiled‐coil oligomers with pLDDT > 70 in both reference sequence and its frameshifted variant.

Thus, all these examples (see also Fig. S6) suggest that in some cases, a frameshift event can give rise to novel structures and functions. This arrangement of the transcripts provides the basis for the synthesis of multiple protein variants within the same coding region, with each variant potentially being produced as needed by the cell. As a result of this analysis, some of the frameshifted structures were selected for future experimental evaluation of the predicted structures.

### Propensity of TR regions to aggregate

Some proteins tend to aggregate. In certain cases, these aggregates play functional roles [33], and in others, they are linked to amyloidosis or other diseases [34, 35]. TR regions of several reference proteins, such as Huntingtin, α‐synuclein, and yeast prions, form aggregates. It was interesting to test how the frameshift in TRs changes their aggregation potential. For this purpose, we used ArchCandy predictor [36]. This choice was made because most other predictors [37] primarily favor hydrophobic residues within aggregation‐prone regions (ARs), whereas ArchCandy also accounts for the high aggregation potential of Gln‐ and Asn‐rich regions. We focused on the eukaryotic sets because the limited number of TRs with ARs in prokaryotic sequences renders them unsuitable for statistical tests.

**Fig. 6:**
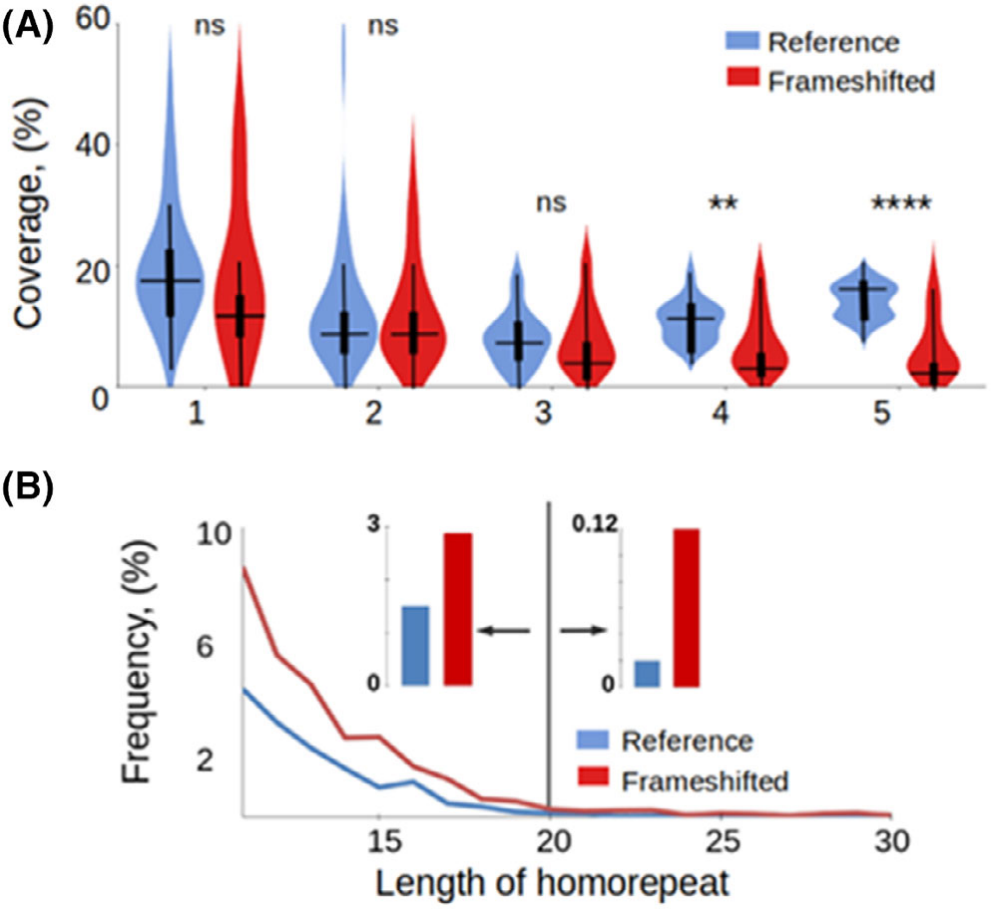
Amyloidogenic region (AR) coverage and hydrophobic homorepeat distributions in reference and frameshifted eukaryotic sequences. (A) Coverage of ARs within tandem repeat (TR) regions of the proteins in eukaryotic organisms predicted by ArchCandy. Results for reference sequences are in blue and for frameshifted ones in red. In the violin plots, the horizontal line marks the median, and the thick vertical bar denotes the interquartile range (25th–75th percentiles). The coverage of ARs in TR regions was calculated by summing up all predicted AR residues overlapping with TR regions divided by the total number of amino acids in the TR regions of the set. The observed variance performed by the two‐sided *T*‐test is statistically significant in TR groups, except group 5. **** and ** mean *P*‐value < 0.0001 and *P*‐value < 0.01, respectively. ns means non‐significant. (B) Length distribution of hydrophobic homorepeats (Ile, Phe, Val, Leu, Trp, Met, Ala, Tyr) in reference and frameshifted sets of eukaryotes. The values on the graph are calculated as the number of hydrophobic homorepeats of a certain length divided by the total number of all detected homorepeats. The vertical line subdivides the homorepeats into those that have high aggregation potential (lengths from 11 to 20 residues) and homorepeats able to form transmembrane helices (from 21 and more). The insets on both sides of the vertical line show the difference between the average frequencies of the reference and frameshifted homorepeats within these two length ranges.

Overall, ArchCandy predicted a steady decrease in AR coverage with increasing repeat length for both reference and frameshifted TRs (Fig. 6A). In short TRs (groups 1–3), reference TRs are slightly more aggregation‐prone than frameshifted ones, although this difference is not statistically significant. The difference becomes more pronounced and statistically significant in TRs with longer repeats (groups 4 and 5). Given that the short frameshifted repeats exhibit higher hydrophobicity than the reference short repeats, the lower aggregation potential of short repeats in the frameshifted set appears unexpected. This result can be attributed to the higher frequency of Gln‐ and Asn‐rich regions in the short reference repeats (groups 1–3). For example, poly‐Gln is one of the most frequent reference homorepeats in eukaryotes [20] (Fig. 4A and Table S1).

Thus, our analysis revealed a general trend: frameshifted TR sequences are less aggregation‐prone than reference sequences. This suggests that evolutionary selection may reduce the harmful effects of frameshift mutations.

### Implications of hydrophobic homorepeat relocalization during frameshifting events

If homorepeats consist of hydrophobic AA, they tend to aggregate. However, when the length of a homorepeat exceeds 20 residues, it can be inserted into the membrane, form transmembrane (TM) α‐helices, and avoid aggregation [26]. We analyzed the frequency of hydrophobic homorepeats depending on their length and found that, in general, frameshifted sequences have more hydrophobic homorepeats than reference sequences (Fig. 6B). This is especially true for homorepeats exceeding 20 residues with the potential to form TM helices. We observed that often during a frameshift, either hydrophobic homorepeats in the reference sequence turn into hydrophilic ones, or, conversely, hydrophilic ones turn into hydrophobic ones. Consequently, frameshifting in the homorepeat region can alter the solubility or insolubility of certain proteins and (or) their localization, whether within the membrane or elsewhere. Given that in general the frameshift increases the number of hydrophobic homorepeats, it should lead to an increase in aggregation and location in the membranes.

## Discussion

TRs can undergo amplification or deletion through simple genetic mechanisms, such as replication slippage or homologous recombination, enabling rapid evolution of new sequences and making them fertile ground for structural and functional innovation [23, 24, 25]. Frameshift mutations, minor nucleotide‐level changes that can result in major alterations at the protein level, are another potent source of variation driving evolutionary selection. The convergence of these two processes is particularly intriguing, as TRs are known hotspots for frameshift events [21, 22]. This motivated us to systematically investigate how frameshifts within TR regions might drive sequence variation, alter structural properties, and give rise to novel functional outcomes.

Our large‐scale comparison showed that the disparity in physicochemical characteristics between reference and frameshifted TR sequences is greatly influenced by repeat unit length. Shorter repeats exhibit more pronounced differences, whereas repeats of 50 residues or longer tend to converge with their characteristics resembling both each other and the average values found across the entire proteome (Figs 3, 4 and 6). Short repeats in frameshifted sequences exhibit increased hydrophobicity and a reduced number of IDRs. The newly emerged hydrophobic regions generated by frameshifts in TRs may serve as a source of new aggregation‐prone regions and contribute to various pathologies. This finding is particularly interesting given previous work [17], which concluded that, overall, frameshifted sequences contain fewer hydrophobic regions and more IDRs than their reference counterparts. It is important to note that the reduction in IDRs we observed occurs specifically in regions with short repeat units (groups 1–3), rather than uniformly across all frameshifted sequences. Previous studies have suggested that such short TRs in reference sequences are subject to evolutionary pressure to maintain high hydrophilicity [20, 28]. Thus, our observation is more likely explained by selection for high hydrophilicity in the short reference TRs, rather than by selection for hydrophobicity in their frameshifted TRs.

We found that frameshifted sequences contain a greater number of hydrophobic homorepeats, which may contribute to an increased number of aggregation‐prone regions and transmembrane helices compared to reference sequences. In numerous instances, frameshifting in the homorepeats can alter the solubility or insolubility of certain proteins and (or) their localization, whether within the membrane or elsewhere. This is particularly important, as homorepeats frequently occur in rapidly evolving, essential proteins that are crucial for organismal survival [38]. The transformation of these homorepeats may thus promote rapid adaptability.

In regard to AA composition, the most prominent difference is a substantially higher percentage of Arg in the frameshifted TRs than in the reference ones, being approximately three times greater in eukaryotes and about seven times higher in prokaryotes. Among the most frequent repetitive motifs of the frameshifted sequences are poly‐Arg and dipeptide repeats Gly‐Arg and Pro‐Arg. A question arises: what is the reason for this anomaly? Analysis of homorepeats suggests one possible explanation. At the DNA level, homorepeats are often encoded by repeated use of the same codon. In eukaryotic sequences, CAG (Gln) is the most frequently used, followed by eight codons with > 4% frequency: GGC (Gly), GAG (Glu), TCC (Ser), GCC and GCG (Ala), CCG and CCA (Pro), and CAA (Gln) (Fig. S5). The remarkable increase in poly‐Arg within the frameshifted homorepeats may be explained by the fact that Arg is encoded by six different codons, three of which, in the main frame, encode for Glu, Ser, and Ala that are abundant in the reference homorepeats (Figs S4 and S5). The other AAs (Ser and Leu) encoded by six different codons in the frameshifted homorepeats increase to a lesser extent due to the lower frequencies of the corresponding codons in the reference homorepeats (Figs S3–S5).

In the reference proteins, Arg‐rich repeats play various functional roles. For example, Arg‐rich intracellular delivery (AID) peptides transport big molecules into cells in plants using both covalent and noncovalent protein transductions [39]. The C‐terminal part of U1‐70K protein, a small nuclear ribonucleoprotein particle, which contains an Arg‐Ser dipeptides region, may be involved in the regulation of mRNA splicing in Drosophila and pre‐mRNA splicing in plants [40, 41]. Mammalian heterogeneous nuclear ribonucleoprotein (hnRNP) E1B‐AP5 is methylated *in vivo* within Arg‐Gly‐Gly (RGG)‐box motifs. E1B‐AP5 is recognized for its role in mediating protein‐RNA interactions [42]. It has also been also found that Arg‐rich proteins can form liquid‐like droplets through liquid–liquid phase separation (LLPS). Arg can switch between dense and liquid phases at high salt concentrations, but not lysine [43]. Thus, Arg may be used to control droplet behavior [43]. We found that Arg‐containing two‐residue TRs are especially frequent in the reference proteins. A number of studies suggest that these TRs have specific functions, being involved in protein binding to DNA and RNA, participating in the organization of an extracellular matrix, and being important elements of the LLPS process [44]. For example, Arg‐Ser dipeptide repeats are involved in alternative splicing control, Arg‐Gly in transcriptional regulation RNA binding, Arg‐Val regions in gene carriers, and Arg‐Glu in cell survival signaling [45]. The functional significance of these regions in the reference proteins suggests that frameshifted Arg‐rich TRs, once they arise, may confer new functions to the protein.

An imbalanced increase of Arg‐rich TRs in frameshifted sequences may also have a significant pathological impact. It has been shown that Arg‐rich sequences are highly toxic in cell and animal models of amyotrophic lateral sclerosis (ALS) [46, 47, 48]. The same dipeptides may affect ribosome‐associated quality control [49]. Furthermore, Pro‐ and Arg‐rich peptides have been demonstrated as allosteric inhibitors of the 20S proteasome [50]. Arg‐rich peptides are also modulators of protein aggregation and cytotoxicity, playing a role in Alzheimer's disease [51]. In addition, *de novo* frameshifts in HMGB1 protein, which replace the intrinsically disordered acidic tail of HMGB1 with an Arg‐rich basic tail, cause brachyphalangy, polydactyly, and tibial aplasia/hypoplasia syndrome (BPTAS) [52]. Thus, our finding of the exceptionally high abundance of Arg as a result of frameshifting in TRs points out to a potential link of the frameshifts with pathological effects.

Other frequent short repeats identified in frameshifted sequences, lacking Arg, are also known to play important functional roles in reference proteins. For example, Gly‐Ser repeats in chimeric antigen receptor (CAR) linkers provide the flexibility necessary for antigen‐binding sites to change conformation [53].

An important property of protein sequences is their potential to aggregate, which can either serve functional roles or contribute to diseases such as amyloidosis. Since mutations can promote unwanted aggregation, we investigated how frameshifting in TRs affects their aggregation potential. Our analysis (Fig. 6A) revealed that, overall, frameshifted TRs are less aggregation‐prone than reference sequences, suggesting evolutionary selection against harmful frameshift effects.

The development of alphafold opens new avenues for large‐scale modeling of protein structures. Previously, we modeled structures of protein isoforms with frameshifts occurring within one or a few long repeats in a TR structural domain. alphafold2 modeling suggested that these frameshifts do not disrupt the overall structure of the domains composed of TRs. The frameshifted repeat fits into the remaining structure [31]. In the present study, we used alphafold2 to perform large‐scale modeling of structures composed entirely of frameshifted TRs. The vast majority of the models had low confidence scores. This was especially true for the long repeats belonging to classes III, IV, and V of the structural classification [18]. Among the TR regions formed by short repeats from Class II, we found several alphafold2 models with high confidence scores featuring either a collagen triple helix or α‐helical coiled‐coil oligomers. Thus, our large‐scale alphafold modeling suggests that frameshift events can generate novel structures, enabling the production of multiple protein variants from the same coding region. A subset of the most interesting frameshifted structural models was chosen for subsequent experimental evaluation, and the results will be reported upon completion.

Thus, our analysis revealed that frameshift can have distinct and significantly stronger effects on TR regions compared to non‐repetitive sequences. While previous studies have indicated that, despite frameshift causing differences in protein sequences, several of their characteristics, including their hydrophobicity profiles and intrinsic disorder profiles, maintain similarity in the corresponding frameshifted sequences [17], our current study demonstrates that frameshifts in TR regions, particularly those composed of short repeats, lead to drastic changes. These changes include increased hydrophobicity, Arg‐rich sequences, novel aggregation‐prone regions, and transmembrane helices. Consequently, frameshifts in TRs may be most crucial for altering binding partners, cellular localization, and other functional roles. Moreover, these frameshifts can pose significant risks and contribute to various pathologies. Our *in silico* study of amino acid sequences paves the way for computational screening of frameshift‐promoting nucleotide motifs around TRs, along with the analysis of existing RNA‐seq and mass spectrometry data, to further assess their likelihood of *in vivo* expression. Detected frameshifted TRs with the highest structural and functional potential can be experimentally validated and their structures resolved at atomic resolution.

## Materials and methods

### Selection of datasets

We selected coding sequences (CDSs) of 50 well‐studied organisms (21 prokaryotes and 29 eukaryotes) from RefSeq and Ensembl databases [54] (Fig. 1 and Fig. S1). Then, we obtained a non‐redundant set of CDSs to avoid duplicated sequences coming from RefSeq and Ensembl databases by clustering the sequences with CD‐HIT (sequence identity of 100%) [55]. The reference set includes protein sequences obtained from the translation of the CDSs in the main reading frame. The frameshifted set includes amino acid (AA) sequences obtained from the translation of CDSs in two frames (−1 and +1). The minimal length of the frameshifted sequence was set at 60 AA. As a result, the reference set had 1 032 420 sequences and the frameshifted set had 3 005 214 sequences.

### Prediction of structural states of reference and frameshifted sequences

In AA sequences, we predicted regions with Pfam, CATH domains, short linear motifs (SLiMs) by the tapass pipeline [26]. Aggregation‐prone regions (ARs) and exposed aggregation‐prone regions (EARs) were predicted by archcandy 2.0 [36] and tango [37]. Intrinsically disordered regions were determined by IUPred [30]. The 3D structures were predicted by the alphafold2 and alphafold3 programs [27].

### Prediction of TR regions in reference and frameshifted sequences

TRs were predicted by MetaRepeatFinder, which employed multiple TR finders, each excelling within a specific range of repeat lengths (https://bioinfo.crbm.cnrs.fr/index.php?route=tools&tool=15). In the MetaRepeatFinder pipeline, Selectseq and Regex finders predict TRs with repeat units of 1 to 3 AA, T‐REKS [56] is the most suitable for the identification of TRs with units until approximately 15 AA in length, and TRUST [57] for repeats longer than 15 residues. A software package of tools, incorporating programs for sensitive generalized sequence profiling [58], was used to detect collagen‐like and α‐helical coiled‐coil TRs in protein sequences.

Raw data from the structural state predictions of both reference and frameshifted TR sequences are provided in: https://zenodo.org/records/16083092.

### Clustering of TRs

We used an in‐house script for the clustering of TRs. The clustering was based on TR consensus motifs. Consensus sequences of the multiple sequence alignment of TRs were retrieved using an algorithm designed for T‐REKS [56]. The values of each AA frequency were stored in an ordered vector with a constant AA position. These vectors were then utilized as input data for clustering using the DBSCAN algorithm [59].

### Codon usage in different reading frames of homorepeats

We calculated codon usage in the DNA regions where at least 9 AA of the reference reading frame overlap with frameshifted homorepeat regions. For comparison, we also calculated the codon usage in all proteins of the analyzed proteomes. The analysis was done by an in‐house script. The following formula was used for computing the frequency of codons that are conserved within TR regions: Frequency of conserved codons = Number of conserved codons/(number of conserved codons + number of variable codons) × 100%, where codons were considered to be variable if at least one mutation at the DNA level was detected within a TR region.

### alphafold modeling of conserved structured TR regions from the frameshifted set

We developed in‐house scripts to select TR‐containing frameshifted sequences for alphafold2 structural prediction. In the first step, we searched for sequence similarity within frameshifted sets of different studied proteomes from eukaryotes and prokaryotes separately. To find conserved regions of frameshifted sequences, we ran the blast program with the following parameters: sequence coverage of more than 90%, alignment length ≥ 30 AA, e‐value = 10^−5^, PAM30 as an AA substitution matrix [60]. We focused on five proteomes with the highest number of frameshifted sequences (*Homo sapiens*, *Pan troglodytes*, *Canis lupus*, *Bos taurus*, *Mus musculus*). We ran the TR‐containing frameshifted sequences from each of these proteomes against the remaining 28 frameshifted sets of eukaryotic proteomes. Those frameshifted sequences having regions with at least 1 hit found across 28 eukaryotic species were selected as ‘conserved’ for further analysis. Next, a filter was applied to find the conserved regions, which completely overlap with TR regions. Subsequently, only those sequences that overlapped with structured regions detected by the tapass pipeline were selected and then clustered by CD‐HIT to eliminate redundancy. Finally, this non‐redundant set of the frameshifted sequences was used as input for alphafold2 modeling by using the Jean‐Zay supercomputer at the National Computing Centre for the CNRS (IDRIS, the link to access http://www.idris.fr/eng/info/missions‐eng.html) with default parameters. Based on the algorithm used in tapass [26], we created a filter to distinguish alphafold2 models of ordered regions longer than 20 AAs from those with disordered conformations. The ordered TR regions of alphafold2 models having the confidence level plDDT ≥ 70 were selected for further manual analysis. TRs with repeat units shorter than six residues, classified as Class II according to the structural classification [18], represent a special case. These TRs primarily form two types of structures: collagen triple helices and α‐helical coiled‐coil structures. We identified them in protein sequences by sensitive generalized sequence profiling [58]. They typically form stable 3D structures through self‐association into oligomers, most commonly dimers or trimers. Accordingly, we performed modeling of these TRs in monomeric, dimeric, trimeric, and tetrameric forms. Our tests showed that alphafold3 (AF3) produced better results than alphafold2 for collagen‐like structures; therefore, we used alphafold3 to model these structures.

## Conflict of interest

The authors declare no conflict of interest.

## Author contributions

Conceptualization, AVK, ZO, and IS; methodology, ZO, AVK, TF, and JL; software, ZO, TF, and JL; validation, GA and ZO; data curation, GA, ZO, SMGB, DNPG; data analysis, ZO, SMGB, DNPG, and AVO; writing – original draft preparation, ZO and AVK; writing – review and editing, AVK, ZO, GA, and AVO; supervision, AVK and IS.

## Supporting information


**Fig. S1.** A phylogenetic tree of 50 species that were selected for the analysis.
**Fig. S2.** Frequencies of AAs in all reference proteins (non‐repetitive and repetitive) and in homorepeats.
**Fig. S3.** Codon usage in all reference proteins and homorepeats.
**Fig. S4.** Codon usage in all +1 shifted reference proteins and +1 shifted homorepeats.
**Fig. S5.** Codon usage in all −1 shifted reference proteins (blue) and −1 shifted homorepeats.
**Fig. S6.** alphafold confidence metrics were used to assess the reliability of the structural models in Fig. 5.
**Table S1.** Coverage of AA in TR groups in reference and frameshifted sequences of eukaryotes.
**Table S2.** Coverage of AA in TR groups in reference and frameshifted sequences of prokaryotes.
**Table S3.** Examples of existing human proteins whose frameshifted sequences contain known protein domains.

## Data Availability

Raw data from the structural state predictions of both reference and frameshifted TR sequences can be accessed via the following link: https://zenodo.org/records/16083092.
