# Supplementary material for: Protein tandem repeats that produce frameshifts can generate new structural states and functions
Source: FEBS J. 2025 Sep 24;293(3):842–58. doi: 10.1111/febs.70273 (PMC12871928; doi:10.1111/febs.70273)
Supplement: Supplementary file 1 — Fig. S1. A phylogenetic tree of 50 species that were selected for the analysis. Fig. S2. Frequencies of AAs in all reference proteins (non‐repetitive and repetitive) and in homorepeats. Fig. S3. Codon usage in all reference proteins and homorepeats. Fig. S4. Codon usage in all +1 shifted reference proteins and +1 shifted homorepeats. Fig. S5. Codon usage in all −1 shifted reference proteins (blue) and −1 shifted homorepeats. Fig. S6. alphafold confidence metrics were used to assess the reliability of the structural models in Fig. 5. Table S1. Coverage of AA in TR groups in reference and frameshifted sequences of eukaryotes. Table S2. Coverage of AA in TR groups in reference and frameshifted sequences of prokaryotes. Table S3. Examples of existing human proteins whose frameshifted sequences contain known protein domains. [file FEBS-293-842-s001.pdf]

SUPPLEMENTARY TABLES AND FIGURES

Protein tandem repeats that produce frameshifts can generate new structural states and functions. Osmanli et al. 2025

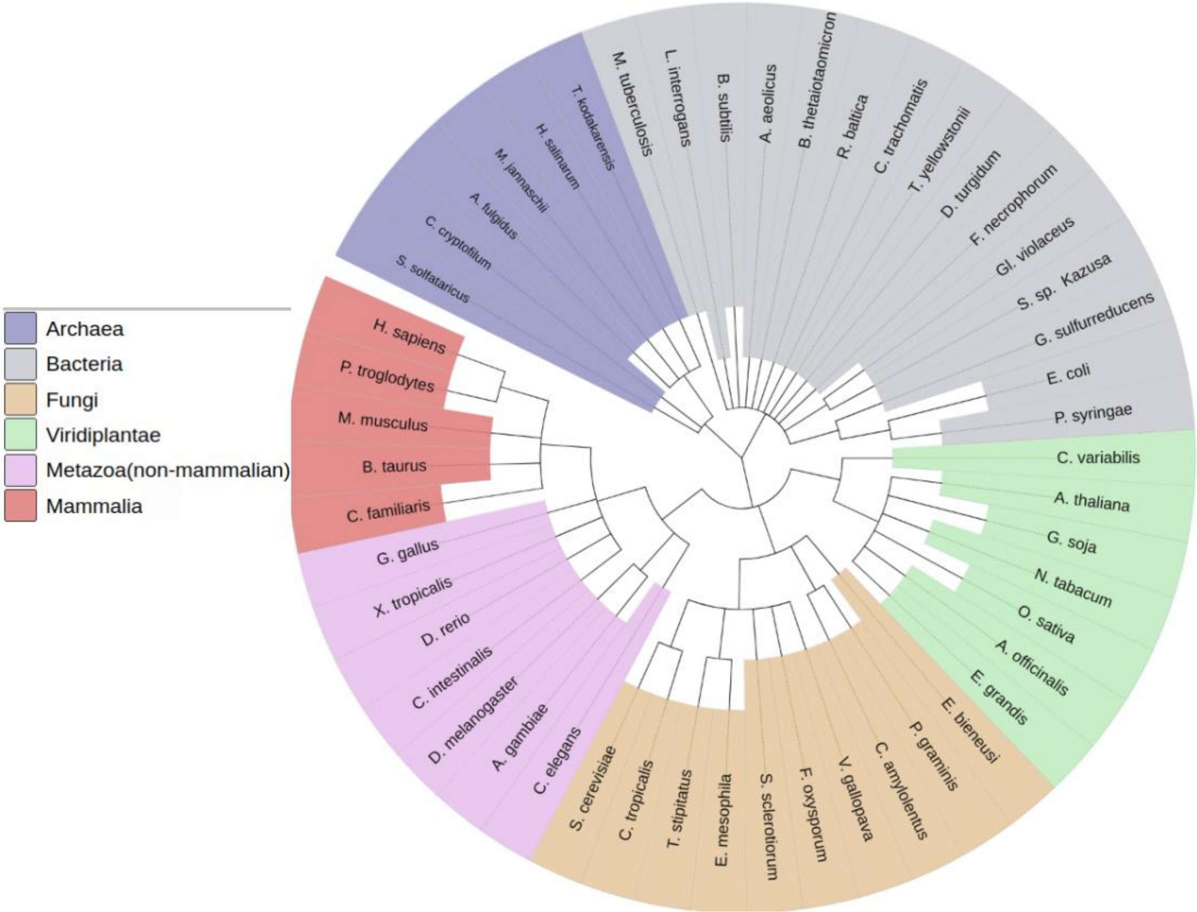

Figure S1. A phylogenetic tree of 50 species that were selected for the analysis. The tree is generated by iTOL.

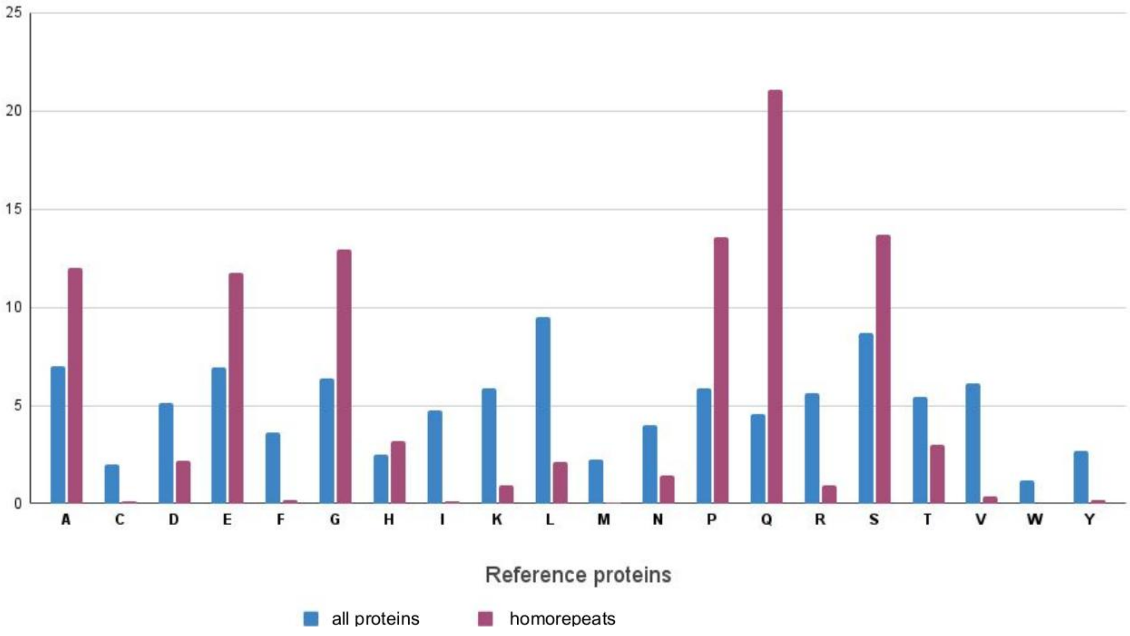

Figure S2. Frequencies of AAs in all reference proteins (non-repetitive and repetitive) (in blue) and in homorepeats (in red).

Table S1. Coverage of AA in TR groups in reference and frameshifted sequences of eukaryotes

	Coverage of amino acids in tandem repeat groups											
	Eukaryotes reference sequences						Eukaryotes frameshifted sequences					
Amino acid	group 1	group 2	group 3	group 4	group 5	mean of groups	group 1	group 2	group 3	group 4	group 5	mean of groups
Ala	12.26	8.34	7.13	6.64	6.74	8.22	22.54	10.59	12.55	8.14	10.74	12.91
Cys	0.18	0.85	0.78	2.83	2.23	1.37	0.75	2.33	2.23	3.31	3.08	2.34
Asp	2.72	4.75	4.14	4.86	5.31	4.36	0.28	1.00	1.30	2.16	2.57	1.46
Glu	13.35	9.09	7.71	6.93	6.68	8.75	1.08	2.72	2.15	3.13	3.09	2.43
Phe	0.29	0.79	1.32	3.33	3.88	1.92	0.45	0.76	0.82	2.09	1.41	1.10
Gly	11.72	10.25	17.02	6.66	6.48	10.43	9.34	11.20	9.85	7.40	9.72	9.50
His	3.35	2.68	1.49	3.19	2.47	2.63	1.23	3.46	2.80	4.11	4.53	3.23
Ile	0.23	1.13	2.23	4.54	5.23	2.67	0.69	1.57	1.36	2.90	1.84	1.67
Lys	1.41	4.95	5.44	6.45	5.74	4.80	1.12	1.79	2.14	2.97	1.95	1.99
Leu	2.64	4.58	6.03	9.76	9.61	6.52	5.01	7.72	7.20	9.61	8.94	7.70
Met	0.23	0.75	1.26	1.92	2.06	1.24	0.60	0.92	1.17	1.82	1.27	1.16
Asn	1.65	2.01	1.97	4.47	4.13	2.85	0.87	1.38	1.49	2.32	1.36	1.48
Pro	12.34	11.66	14.00	5.46	5.26	9.74	11.58	14.95	13.20	9.67	10.64	12.01
Gln	18.68	5.04	5.02	4.47	4.26	7.49	2.28	3.49	3.44	4.78	4.83	3.76
Arg	1.06	10.75	5.75	5.31	5.18	5.61	26.72	14.55	15.68	11.23	13.12	16.26
Ser	13.48	13.83	8.85	8.35	8.10	10.52	10.06	9.69	11.24	9.84	8.08	9.78
Thr	3.43	4.35	5.05	5.73	5.68	4.85	4.08	7.00	6.05	6.15	5.07	5.67
Val	0.70	3.11	3.58	5.58	6.68	3.93	0.88	2.95	2.94	4.75	4.83	3.27
Trp	0.05	0.27	0.22	0.90	1.33	0.55	0.32	1.42	1.80	2.47	2.13	1.63
Tyr	0.26	0.84	1.03	2.61	2.95	1.54	0.15	0.54	0.60	1.16	0.81	0.65

Table S2. Coverage of AA in TR groups in reference and frameshifted sequences of prokaryotes

	Coverage of amino acids in tandem repeat groups											
	Prokaryotes reference sequences						Prokaryotes frameshifted sequences					
Amino acid	group 1	group 2	group 3	group 4	group 5	mean of groups	group 1	group 2	group 3	group 4	group 5	mean of groups
Ala	5.74	11.17	13.42	8.49	8.67	9.50	1.39	7.93	11.06	9.37	10.55	8.06
Cys	0.23	0.21	0.48	1.01	0.95	0.58	0.37	2.38	2.16	3.68	3.01	2.32
Asp	3.28	11.97	4.03	5.52	5.56	6.07	0.74	2.11	1.91	3.20	3.90	2.37
Glu	8.56	6.21	6.86	7.31	6.68	7.12	0.46	0.86	1.10	1.98	1.86	1.25
Phe	0.23	1.88	2.25	4.07	4.22	2.53	0.00	0.79	0.85	2.18	1.68	1.10
Gly	30.60	10.71	19.37	7.75	7.72	15.23	4.73	11.62	10.66	9.71	11.97	9.73
His	1.17	1.72	0.85	1.82	1.97	1.51	0.74	3.73	3.46	3.98	5.08	3.40
Ile	0.35	3.64	4.21	6.70	6.55	4.29	0.09	1.05	0.91	2.09	1.50	1.13
Lys	2.93	3.86	4.63	5.82	5.18	4.48	0.46	0.87	1.34	2.17	1.25	1.22
Leu	0.47	10.12	8.87	10.23	10.18	7.97	0.46	3.85	3.64	5.92	6.25	4.03
Met	0.35	1.62	1.27	1.93	2.06	1.45	0.00	0.49	0.43	0.78	0.42	0.42
Asn	0.70	1.88	3.42	4.01	3.79	2.76	0.09	1.16	1.17	1.88	1.31	1.12
Pro	13.83	10.02	4.69	4.09	4.47	7.42	0.93	9.75	10.41	9.47	10.07	8.12
Gln	3.75	2.38	3.24	3.35	3.35	3.21	1.02	2.65	2.82	3.64	3.97	2.82
Arg	3.52	3.45	4.06	5.44	5.47	4.39	77.94	30.61	26.91	17.68	19.15	34.46
Ser	15.36	6.05	6.09	6.04	6.03	7.91	9.27	10.19	9.96	9.39	7.09	9.18
Thr	8.09	6.50	5.04	5.02	5.30	5.99	0.28	6.01	6.71	5.58	4.23	4.56
Val	0.23	5.14	5.53	7.15	7.29	5.07	0.56	2.35	2.76	3.96	4.32	2.79
Trp	0.12	0.33	0.44	1.07	1.25	0.64	0.37	1.27	1.32	2.34	1.59	1.38
Tyr	0.47	1.14	1.24	3.20	3.31	1.87	0.09	0.35	0.45	1.03	0.81	0.54

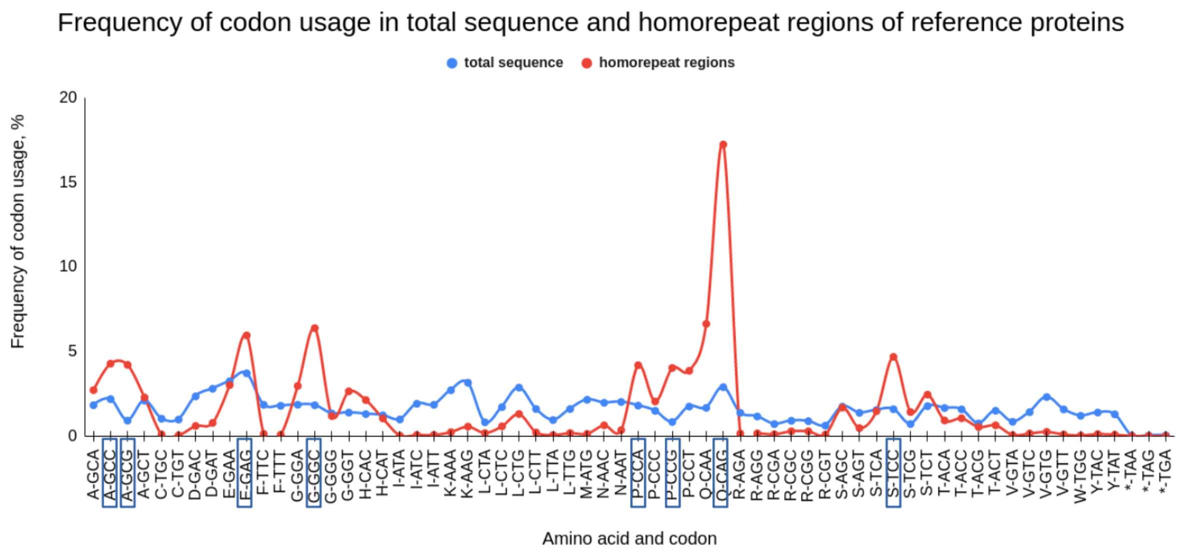

Figure S3. Codon usage in all reference proteins (blue) and homorepeats (red). The most frequent codons in homorepeats are marked by rectangles. The largest peak is at CAG codon (poly-Gln).

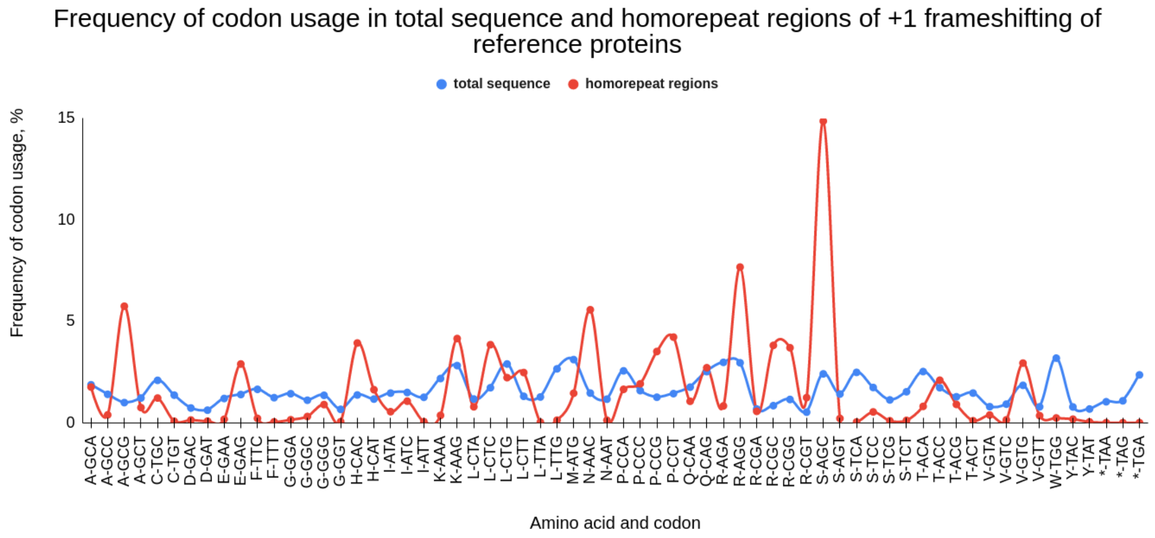

Figure S4. Codon usage in all +1 shifted reference proteins and +1 shifted homorepeats (red).

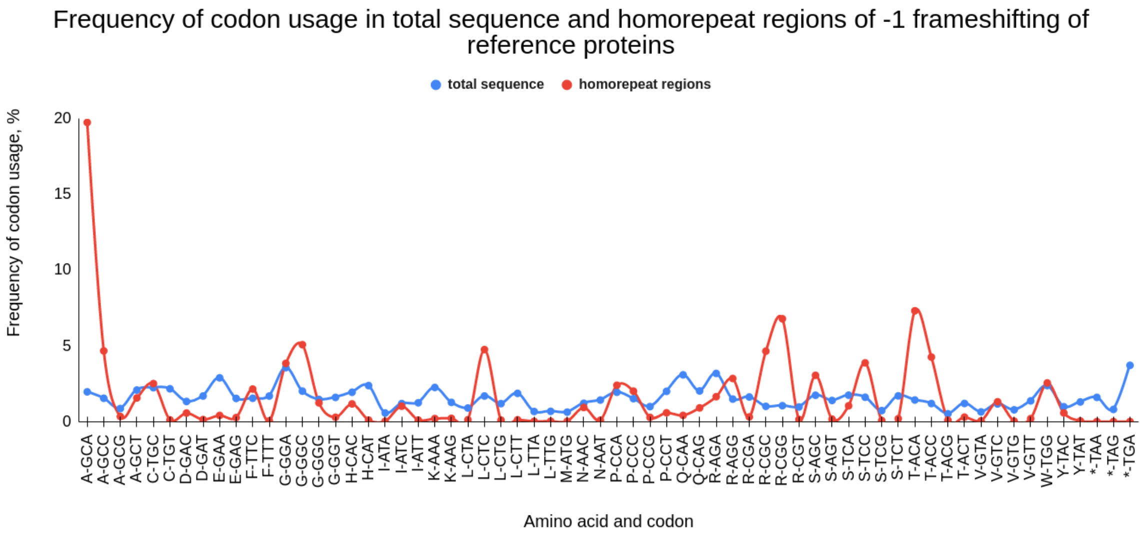

Figure S5. Codon usage in all -1 shifted reference proteins (blue) and -1 shifted homorepeats (red).

Table S3 Examples of Existing Human Proteins Whose Frameshifted Sequences Contain Known Protein Domains

Zinc fingers in frameshift sequence

Frameshift sequence ID	UniProt ID	Protein name	MS identified peptides*	Transcript support level**
ENST00000439326.8_ORF-F-3|379-640|262	Q08AG5	ZNF 844	✓	TSL1
NP_001121695.1_ORF-F-3|650-747|98	Q9BY31	ZNF 717	✓	curated mRNA
ENST00000315678.10_ORF-F-2|8-347|340	Q8N446	ZNF 843	-	TSL2
ENST00000414399.1_ORF-F-2|0-106|107	C9J092	ZNF 668	-	TSL3
ENST00000456748.6_ORF-F-3|246-437|192	G3V0F4	ZSCAN 21	-	TSL5
ENST00000574907.5_ORF-F-3|52-188|137	I3L3B3	XIAP	-	TSL3
ENST00000396150.4_ORF-F-3|0-209|210	Q9NXT0-**2**	ZNF 586	-	TSL1
ENST00000423015.5_ORF-F-3|142-229|88	O75840-**3**	KLF 7	-	TSL1
ENST00000591537.5_ORF-F-3|77-246|170	Q9BX82-**2**	ZNF 471	-	TSL2
NP_001308046.1_ORF-F-3|57-332|276	-	ZSCAN 22	-	curated mRNA

ZSCAN = Zinc finger and SCAN domain containing protein
XIAP = XIAP associated factor 1
KLF = Krueppel-like transcription factor

Ankyrin repeats in frameshift sequence

Frameshift sequence ID	UniProt ID	Protein name	MS identified peptides*	Transcript support level**
ENST00000427211.3_ORF-F-3|36-173|138	E5RJM6-**2**	ANKRD 65	-	TSL1
ENST00000579755.2_ORF-F-2|55-131|77	Q8N726	CDKN2A 214<br>Tumor suppressor ARF	✓	TSL1

ANKRD = ankyrin repeat domain
CDKN2A = cyclin dependent kinase inhibitor 2A
* mass-spectral data from UniProt database
** TSL = Transcript support level from Ensembl database

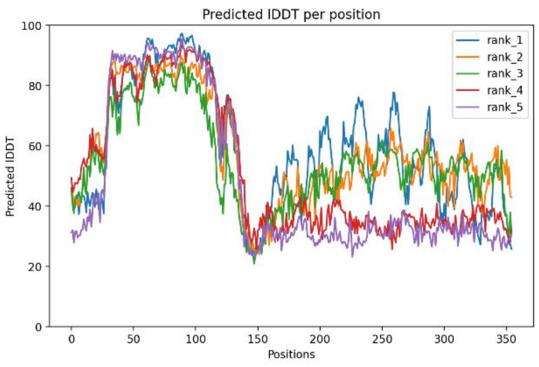

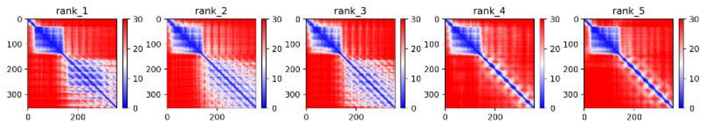

Zinc finger protein 781 (ZN781_HUMAN)
https://www.modelarchive.org/doi/10.5452/ma-s50pj

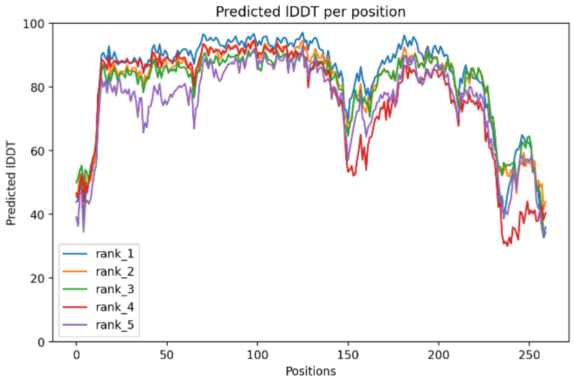

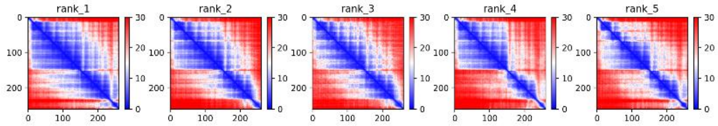

Zinc finger protein 781 (ZN781_HUMAN), frameshifted sequence
. https://www.modelarchive.org/doi/10.5452/ma-mx4gi

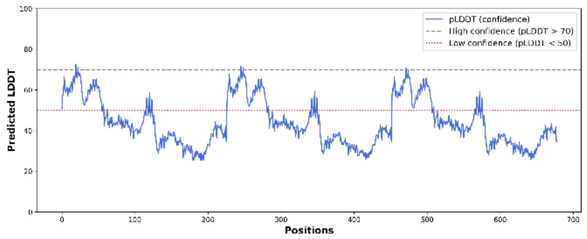

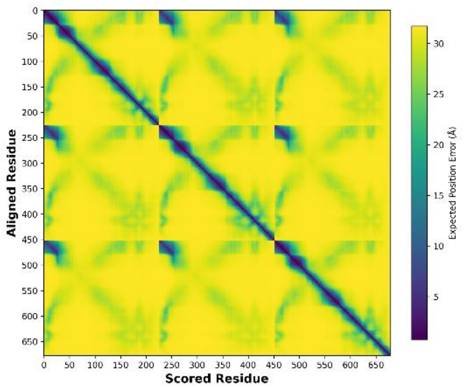

Uncharacterized protein LOC563353 from *Danio rerio* (Q1LYI5_DANRE).
https://www.modelarchive.org/doi/10.5452/ma-etqym

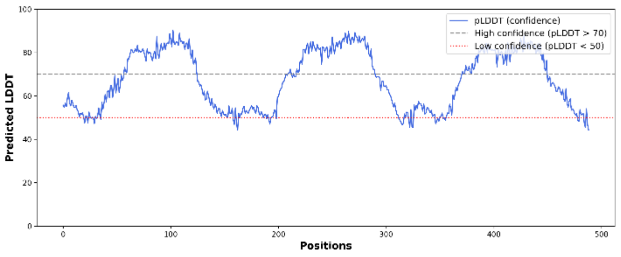

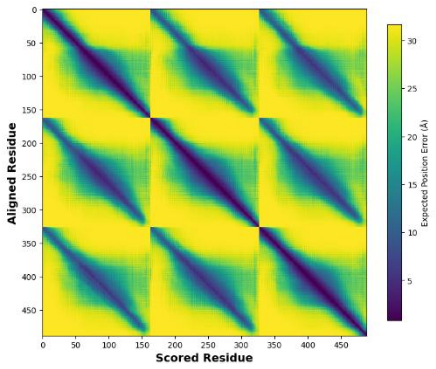

Uncharacterized protein LOC563353 from *Danio rerio* (Q1LYI5_DANRE). Fragment of frameshifted sequence

https://www.modelarchive.org/doi/10.5452/ma-3ysg9

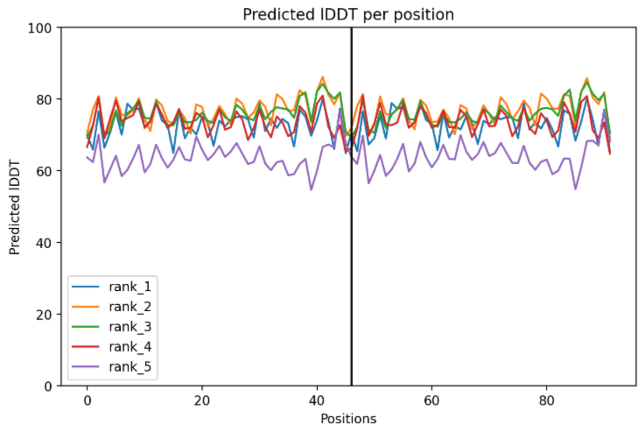

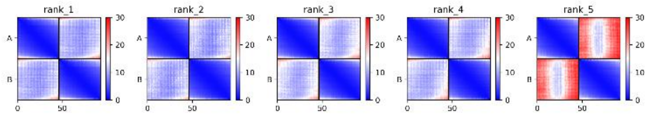

Trophinin (*Mus musculus*) E9Q160_MOUSE. Coiled coil dimer.

https://www.modelarchive.org/doi/10.5452/ma-0op52

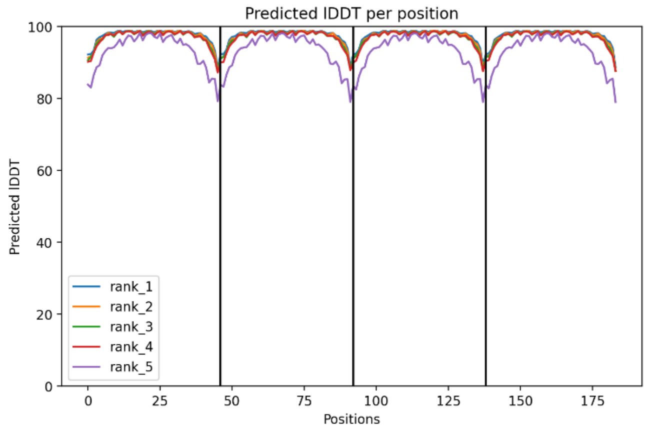

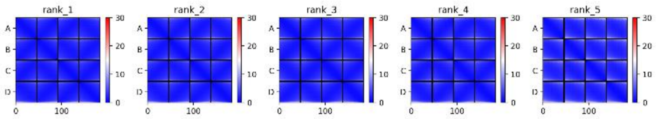

Trophinin (*Mus musculus*) E9Q160_MOUSE. Coiled coil tetramer. A region of a frameshifted sequence.
https://www.modelarchive.org/doi/10.5452/ma-yuzmx

Figure S6. AlphaFold confidence metrics were used to assess the reliability of the structural models in Figure 5. Left: pLDDT scores (0–100) indicate per-residue confidence, with higher values reflecting greater local accuracy. Right: PAE scores (2D matrix) estimate the expected alignment error between residues; lower values indicate higher confidence in their relative positioning.
